# Ultrasound-Assisted Sustainable Processing of Garden Cress Juice: Enhancing Bioactive Compounds and Bioaccessibility through XGBoost Optimization

**DOI:** 10.1021/acsomega.5c08691

**Published:** 2025-11-20

**Authors:** Okan Levent, Mehmet Ali Şimşek, Seydi Yıkmış, Selinay Demirel, Nazan Tokatlı Demirok, Melikenur Türkol, Moneera O. Aljobair, Nazlı Tokatlı, Isam A. Mohamed Ahmed

**Affiliations:** † Department of Food Engineering, Faculty of Engineering, 37520 Inonu University, Malatya 44280, Türkiye; ‡ Department of Computer Technologies, Vocational School of Technical Sciences, Tekirdag Namik Kemal University, Tekirdag 59030, Türkiye; § Department of Food Technology, Tekirdag Namık Kemal University, Tekirdag 59830, Türkiye; ∥ Nutrition and Dietetics, Faculty of Health Sciences, 162334 Tekirdag Namık Kemal University, Tekirdag 59030, Türkiye; ⊥ Department of Sports Health, College of Sports Sciences and Physical Activity, Princess Nourah Bint Abdulrahman University, Riyadh 11671, Saudi Arabia; # Department of Computer Engineering, Faculty of Engineering and Natural Sciences, Istanbul Health and Technology University, Istanbul 34421, Türkiye; ∇ Department of Food Sciences and Nutrition, College of Food and Agricultural Sciences, King Saud University, P.O. Box 2460, Riyadh 11451, Saudi Arabia

## Abstract

This study aimed to improve the functional and nutritional properties of garden cress (*Lepidium sativum*) juice using ultrasound and optimize process parameters by modeling them with advanced machine learning algorithms. Using a Box–Behnken experimental design, the effects of sonication time (8–16 min) and amplitude (60–100%) on total chlorophyll, total phenolic content (TPC), and ferric reducing antioxidant power (FRAP) were investigated. Nonparametric, high-accuracy estimations were made using the XGBoost algorithm. Optimum conditions were determined to be 12 min and 80% amplitude. Under these conditions, TPC (78.44 mg GAE/mL), FRAP (59.80 mg TE/mL), and chlorophyll (7.15 g/100 mL) values were significantly higher than those in control and pasteurized samples (*p* < 0.05). HPLC-DAD analysis showed that ultrasound treatment positively impacted the phenolic profile by increasing the release of quercetin, quercetin derivatives, caffeic acid, and chrysin. GC-MS data revealed that volatile aroma compounds (especially 1-hexanol, benzaldehyde, and cinnamaldehyde) were preserved mainly by ultrasound. In vitro digestion simulation showed that total postdigestion recovery rates in ultrasound-treated samples were 34.96% for TPC, 32.50% for chlorophyll, and 28.81% for FRAP, demonstrating a significant increase in bioaccessibility. PCA and hierarchical clustering analyses confirmed a significant biochemical separation of ultrasound-treated samples. The findings indicate that ultrasound technology is a superior method for preserving bioactive compounds, maintaining the aroma profile, and enhancing bioaccessibility compared to heat treatment. This enables data-driven process design. The developed model showed a strong predictive performance under optimal conditions. However, the study is limited by the relatively small data set used for model training.

## Graphical abstract

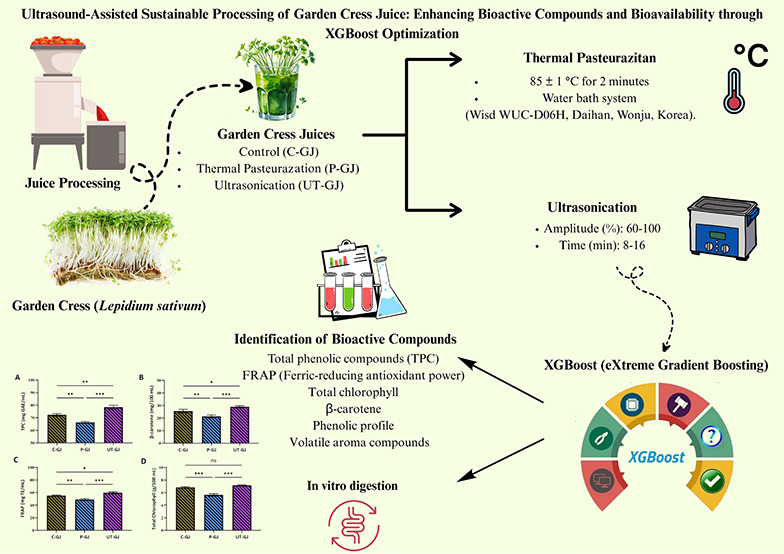

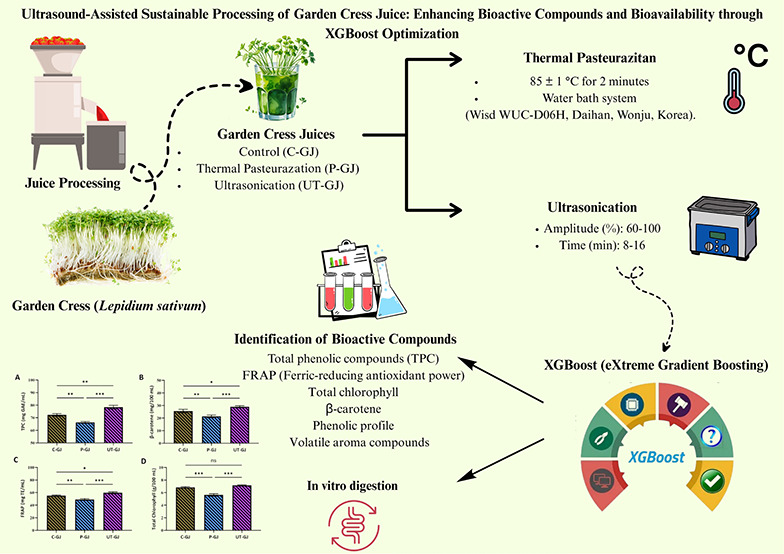

## 1 Introduction

Vegetable juices are perceived as a nutritious and healthy daily drink, and the popularity of consuming fresh vegetables is on the rise.^1^ The potential of cress juice is attributable to its flavor and nutritional value.^2^ Garden cress (*Lepidium sativum*) is a perennial herbaceous plant (i.e., it is capable of growing rapidly) that belongs to the *Cruciferae* (or *Brassicaceae*) family.^3^ The *Brassicaceae* family has been identified as a source of sulfur-organic compounds, such as glucosinolates, which have been shown to have significant health benefits. The improvement of human diets and the prevention of various common diseases can be attributed to these compounds, which have been found to reduce risk factors associated with cardiovascular disease and levels of liver cholesterol.^4^ Since ancient times, various botanical components of the garden cress, including seeds, leaves, roots, and flowers, have been employed in traditional medicine to treat multiple diseases and health complaints.^5^ Garden cress (*Lepidium sativum*), which is characterized by elevated concentrations of fatty acids, proteins, vitamins, and minerals, also contains bioactive components, such as terpenes, kaempferol, glucosinolates, protocatechuic acid, gallic acid, caffeic acid, glucuronide, and coumaric acid.^2^ The latter’s nutritional value is well documented, as are its potential health benefits in fighting malnutrition and promoting overall well-being.^6^

The utilization of conventional food processing techniques has diminished significantly due to the escalating demand for time, thereby unveiling novel prospects for emerging technologies. Ultrasound is one of the most promising nonthermal processing technologies due to its environmental compatibility, safety, and reliability. Ultrasound is a noninvasive, rapid, hopeful, and multifaceted green technology.^7,8^

Ultrasound technology in food processing applications offers versatile advantages such as accelerating mass transfer, reducing process temperatures, increasing extraction efficiency, and ensuring homogeneous distribution of components in heterogeneous systems.^9^ Low-intensity ultrasound (100 kHz–1 MHz, <1 W/cm^2^) is a noninvasive and nondestructive analysis method and is effectively used to monitor physicochemical quality changes that occur in foods during processing and storage.^10^

When evaluating the sensory properties of vegetables, various studies have demonstrated that ultrasound application increases matrix stability and allows for the maximal preservation of bioactive compounds. In particular, ultrasound has been reported to have the potential to stabilize the physicochemical parameters of vegetables and delay spoilage by preserving the structural integrity of polysaccharides, a component of the cell wall.^11^ The nutritional value of garden cress juice is directly related to its bioactive components, such as antioxidant capacity (FRAP), total chlorophyll, and total phenolic compounds. Preserving and enhancing these components are essential for improving the product’s functional properties. Ultrasound applications stand out as a promising method for maintaining the stability and increasing the efficiency of such components. However, the duration of ultrasound and the applied power levels can have varying effects on these components. While the obtained values are directly interpretable, statistical modeling and optimization studies are needed to more clearly and meaningfully demonstrate the impact of process parameters.

In recent years, machine learning (ML) algorithms such as SVM and Random Forest have been widely used for food processing modeling as an alternative to traditional statistical methods.^12,13^ Among these algorithms, Extreme Gradient Boosting (XGBoost) stands out due to its high prediction accuracy, its ability to avoid overfitting, and the advantage of modeling nonlinear relationships. XGBoost has been successfully applied to regression problems for outcomes such as agricultural product quality, extraction yield, and biological activity.^14,15^ Decision trees and ensemble learning methods are combined in XGBoost, an advanced machine learning algorithm. The model’s success is based on its use of gradient-based learning strategies to optimize the loss function.^16^ It is a tree-based method that utilizes distributed and parallel processing, enabling effective performance on large data sets. Balanced performance can be achieved by optimizing the model’s behavior with hyperparameters such as tree depth, number of nodes, learning rate, and subsampling size.^17^ In this study, for the simultaneous estimation of three functional parameters, such as total chlorophyll, total phenolic content (TPC), and antioxidant capacity (FRAP), with a two-factorial experimental design that includes sonication time and amplitude, the XGBoost algorithm based on tree communities was preferred due to its ability to model nonlinear relationships without the need for explicit parameter terms despite its limited sample size.

There is no study in the literature where cress juice ultrasonic process parameters were modeled with XGBoost and optimized for bioactive components. This study aims to model and optimize the ultrasonic process conditions established using the Box–Behnken experimental design, employing XGBoost’s hyperparameter optimization (including tree depth, learning rate, and subsample ratio). Cress juice samples obtained after optimized ultrasonic treatment will be comprehensively characterized for phenolic profiles using HPLC-DAD, bioaccessibility using an in vitro digestion model, and volatile components and sensory analyses using GC-MS. Thus, ultrasonic process optimization aims to maximize the preservation of bioactive components while also ensuring the conservation of functional properties, resulting in a data-driven process design.

## 2 Materials and Methods

### 2.1 Preparation of Garden Cress Juice

Garden cress (*Lepidium sativum*) samples were procured from local agricultural growers in the Tekirdağ region of Türkiye. To preserve their biochemical integrity before analysis, the samples were stored at +4 °C under controlled conditions. In the preparation phase, mature plant parts and stems were carefully separated. The remaining plant material was homogenized using a Waring brand commercial blender (Model HGB2WTS3, United States) to achieve uniform particle size. The resulting mixture was then filtered through Whatman No. 1 filter paper to eliminate fibrous residues. To standardize the macromolecular composition, the filtrate was mixed using a vortex mixer at 2000 rpm for 1 min. The garden cress juice obtained without any further treatment was labeled as the control sample (C-GJ).

### 2.2 Thermal Pasteurization and Ultrasound Treatment

Garden cress juice samples were placed in presterilized 100 mL borosilicate glass containers and pasteurized at 85 ± 1 °C for 2 min in a digital water bath (Wisd WUC-D06H, Daihan Scientific, Korea). The containers were fully immersed to ensure uniform heating, and the temperature was continuously monitored. After gradual cooling to 22 ± 2 °C, the samples were stored at −20 ± 1 °C until analysis. These thermally treated samples were labeled with P-GJ.

Ultrasound treatment was applied to 100 mL juice portions using a Hielscher UP200 St (26 kHz, 200 W, Berlin, Germany) at amplitudes of 60–100% for 8–16 min in continuous mode, with samples kept in an ice bath to prevent overheating. After treatment, they were cooled and stored at −18 ± 1 °C until analysis. Ultrasound-treated samples were coded UT-GJ for subsequent evaluations.

### 2.3 XGBoost (eXtreme Gradient Boosting)

The XGBoost algorithm (eXtreme Gradient Boosting) is an optimized and powerful derivation of the gradient boosting method with various improvements. This algorithm, developed by Chen and Guestrin (2016), has the main advantages of high prediction accuracy, the ability to prevent overfitting, the ability to deal effectively with missing data, and the ability to perform all these operations with high computational efficiency.^18^ XGBoost is a boosting algorithm based on decision trees that combines multiple weak learners into a robust prediction model. At each iteration, the model improves the prediction accuracy by adding a new function to minimize the loss function. A second-order Taylor expansion is applied, which is based not only on the first derivatives (gradient) but also on the information on the second derivative (Hessian). This makes the optimization process more stable and faster.^15,18^ The overall prediction performance of the model is given in eq 1 Each *f*_*k*_ is a decision tree and forms an ensemble model with a total of K trees. The objective function used in the optimization of the model consists of two main components: the loss function *L*(*y*_*i*_,*ŷ*_*i*_), which represents the prediction error, and the regularization term Ω­(*f*_*k*_), which penalizes the complexity of the model.^19^ These equations are given in eqs 2 and 3

1 $${ŷ}_{i}=\varphi \left(x_{i}\right)=\overset{K}{\underset{k=1}{\sum }}f_{k}\left(x_{i}\right)$$

2 $$\mathrm{Obj}\left(\varphi \right)=\underset{i}{\sum }L\left(y_{i},{ŷ}_{i}\right)+\underset{k}{\sum }\Omega \left(f_{k}\right)$$

3 Ω(fk)=γT+12λ∑jwj2

The regularization specified in eq 3 is crucial to prevent overfitting and to increase the generalizability of the model. The regularization term, denoted here as **Ω**(***f**_**k**_*), controls the structural complexity of the model. It applies a penalty of $w_{j}^{2}$ to the number of leaves (***T***) and the squares of the leaf weights (allowing the model to be simplified and better generalized).^18^ This feature makes XGBoost an approach that offers consistently higher accuracy and overall success compared with similar methods such as classical gradient boosting. In this study, the tree-ensemble-based XGBoost model was used for the simultaneous estimation of total chlorophyll, FRAP, and TPC in data from a two-factor experimental design (sonication time and amplitude). XGBoost offers the advantage of learning nonlinear factor interactions without defining additional parameter terms, even with limited sample sizes, and limits overlearning through regularization mechanisms. Compared with other methods evaluated in different application areas (hydrocarbon yield prediction, breast cancer classification, and liquefaction prediction), the XGBoost model demonstrates remarkable superiority with its high prediction accuracy, low error rate, robustness against overfitting, and sensitivity to hyperparameter optimization. Nevertheless, since each algorithm (e.g., ANN, RSM, SVM) has specific strengths depending on data characteristics and problem structure, integrating XGBoost within hybrid or ensemble frameworks can offer enhanced generalization and stability. Therefore, rather than relying on a single model, combining complementary algorithms is likely to yield more balanced and reliable predictive systems in future studies.^20–22^

#### 2.3.1 Hardware and Software Configuration

The computer used to develop the model in this study was equipped with a quad-core Intel64 Family 6 Model 142 Stepping 9 Genuine Intel processor (2.9 GHz) and 7.8 GB RAM. The experimental programming was carried out using Python version 3.10. All modeling procedures were implemented in the Jupyter Notebook environment (version 6.5.4, Python 3.10). The libraries pandas (v1.5.3) and NumPy (v1.23.5) were used for data processing, Matplotlib (v3.7.2) and SHAP (v0.43.0) for visualization, and scikit-learn (v1.3.0) and XGBoost (v1.7.6) for machine learning.

### 2.4 Hyperparameter Selection

The hyperparameters of the model were optimized by grid search with 5-fold cross-validation (GridSearchCV method). This approach was chosen because it is widely used in similar studies in the literature.^15^ The grid search comprised combinations of the number of trees (n_estimators, between 100 and 500), the maximum depth (max_depth, between 2 and 4), and the learning rate (learning_rate, between 0.05 and 0.10). The subsample and colsample_bytree parameters were set to 0.8, a value often recommended in the literature to limit model variance in small data sets.^19^ The root-mean-square error (RMSE) was chosen as the performance measure. The configuration that yielded the lowest average RMSE (250 trees, depth = 3, learning rate = 0.10) was used as the common hyperparameter set for all results.

### 2.5 Total Phenolic Compounds (TPC)

The total phenolic content (TPC) was quantified. This was done according to the Folin–Ciocalteu method.^23^ TPC was determined by extracting 2 mL of each sample with 8 mL of 80% methanol, followed by centrifugation at 4000 rpm for 20 min. A 50 μL aliquot of the supernatant (dilution factor: 5) was mixed with 100 μL of Folin–Ciocalteu reagent and 1500 μL of deionized water. After 10 min of incubation, 50 μL of 20% Na_2_CO_3_ was added. The reaction proceeded in the dark for 2 h, and the absorbance was recorded at 765 nm. Results were expressed as milligrams of gallic acid equivalents (GAE) per 100 mL of sample.

### 2.6 Ferric-Reducing Antioxidant Power (FRAP)

The FRAP assay was performed to assess total antioxidant activity as previously described.^24^ The FRAP working solution was prepared by combining 50 mL of 0.3 M acetate buffer (pH 3.6), 5 mL of 0.01 M 2,4,6-tri­(2-pyridyl)-1,3,5-triazine (TPTZ) solution, and 5 mL of 0.02 M FeCl_3_6H_2_O. The mixture was incubated at 37 °C before use. For the assay, 0.1 mL of the sample was mixed with 4.9 mL of the working solution and incubated at 37 °C for 10 min. Absorbance was recorded at 593 nm. Antioxidant capacity was expressed as millimolar Trolox equivalents (TE) per liter of garden cress juice.

### 2.7 Total Chlorophyll

Chlorophyll was quantified following a dimethyl sulfoxide extraction procedure.^25^ A 3 mL portion of garden cress juice was mixed with 3 mL of acetone (80% v/v) and then filtered. The filtrate’s 645 and 663 nm absorbances were measured, and chlorophyll content was calculated using the following equations:

4 Chlorophylla=(11.85×A663)−(1.54×A645)

5 Chlorophyllb=(21.03×A663)−(5.43×A645)

6 Totalchlorophyll=(chlorophylla)−(chlorophyllb)

### 2.8 β-Carotene

Garden cress juice samples were analyzed using minor modifications of spectroscopic methods to determine their total carotenoid content.^26,27^ An aliquot of 1 mL garden cress juice was extracted with 5 mL methanol (1:2, v/v) and left for phase separation. The upper layer was mixed with 0.5 mL saturated NaCl, shaken, and dried with sodium sulfate. After centrifugation (4000 rpm, 10 min; GYROZEN 1730 R, Korea), the supernatant was re-extracted with 5 mL methanol. Absorbance was measured at 450 nm using a UV–Vis spectrophotometer (SP-UV/vis-300SRB), and carotenoid content was expressed as mg β-carotene/100 mL based on a standard curve.

### 2.9 Identification of Phenolic Profile

Analysis of phenolic compounds was carried out using an Agilent 1260 Infinity HPLC system with a DAD (Diode Array Detector) detector. The separation was carried out at 30 °C on a C18 reversed-phase column of 250 × 4.6 mm with a particle size of 5 μm and a flow rate of 0.80 mL/min. Determinations were made at 280, 320, and 360 nm according to the maximum absorption wavelengths of the compounds. The analyzed phenolic compounds included chlorogenic acid, 4-hydroxybenzoic acid, *o*-coumaric acid, catechin hydrate, hydroxycinnamic acid, quercetin, *p*-coumaric acid, chrysin, trans-ferulic acid, and naringenin. Calibration curves were prepared in the concentration range of 2.5–250 mg/L, and the correlation coefficient (*R* ^2^) for all compounds was found to be above 0.99. The results obtained were reported in μg/mL by taking the average of three parallel analyses.^28^

### 2.10 Volatile Aroma Compounds

The volatile component profiles of garden cress juice samples were analyzed using a Shimadzu gas chromatography–mass spectrometry (GC-MS) system according to the method described by Yıkmış et al. (2021). In the identification of volatile compounds, the retention index (RI) of each compound was calculated under the same analytical conditions, using the *n*-alkane series in the C8–C20 range as a reference. Peak identification was performed by comparing the obtained mass spectra with data from Wiley 7 and NIST 05 mass spectrum libraries. Quantitative analysis was performed using a specific equation based on the relative abundance values of each compound.^29^ Quantification of volatile compounds was performed from relative abundances as follows:

$$C\left(\mu g/kg\right)=\left(C_{is}/A_{is}\right)\times A_{c}\left(\mu g/kg\right)$$

*C*: mean relative abundance; *C*_is_: concentration of internal standard; *A*_is_: peak area of internal standard; and *A*_c_: peak area of the compound.

### 2.11 In Vitro-Simulated Gastrointestinal Digestion Analysis

In the study, an in vitro digestion model was applied according to the protocol developed by Minekus et al. (2014), followed by dialysis. The applied methodology consisted of three consecutive digestion phases: oral (α-amylase, pH 7.0), gastric (pepsin, pH 3.0), and intestinal (pancreatin and fresh bile, pH 7.0). Following the completion of the gastric and intestinal phases, samples were analyzed for total chlorophyll (g/100 mL), total phenolic compound (TPC) content, and antioxidant capacity (by the FRAP method). All analyses were performed in triplicate for each treatment and each replicate, and the results were reported as mean values.^30^

### 2.12 Statistical Analysis

All analyses were conducted in triplicate, and results are reported as mean ± standard deviation (SD). Statistical analysis was performed using one-way analysis of variance (One-Way ANOVA), and significant differences between group means were determined at *p* < 0.05 using Tukey’s HSD (Honest Significant Difference) test. All statistical procedures were performed using SPSS 22.0 software (SPSS Inc., Chicago, IL, USA).

## 3 Results and Discussion

### 3.1 Results of the XGBoost Model

Machine learning techniques, particularly the XGBoost algorithm, accelerate and increase the accuracy of the analytical evaluation of fruit juices, demonstrating the potential of advanced analysis models for rapid and reliable quantification of antioxidant capacity when integrated with electrochemical impedance spectroscopy-based biosensors.^17,31^ This section evaluates the predictive performance of the XGBoost algorithm in modeling the individual effects of two independent variables—ultrasound time and sonication amplitude—on three dependent functional responses: total chlorophyll, antioxidant capacity (FRAP), and total phenolic content (TPC). Separate XGBoost models were trained for each output variable, allowing a specific analysis of the influence of input parameters on each functional metric.

Table 1 contains a comparative analysis of the experimentally determined functional responses and the values predicted by the XGBoost algorithm. The predictions were evaluated for individual conditions as well as for the parameter set optimized by the model for total chlorophyll, FRAP, and TPC outputs. The table (row “% difference”) shows the mean relative error (MRE) for each answer. According to this, very low error rates of 0.38% for total chlorophyll, 1.80% for FRAP, and 0.77% for TPC were determined. This indicates that the model can make very accurate predictions, especially under optimal conditions. Although the highest relative error was observed for the FRAP parameter, the fact that this difference remains below 2% indicates that the model is within practical reliability limits. Suppose one also considers the standard deviations of the experimental data (e.g., ±0.92 mg of TE/ml for FRAP). In that case, it becomes clear that the predictive accuracy of the model is comparable to that of the experimental variation. This speaks in favor of the consistent predictive ability of the model compared to the experimental system.

**Table 1:** Measured and Predicted Responses Used in XGBoost Modelling^a^

	Independent variables		Dependent Variables					
			Total Chlorophyll (g/100 mL)		FRAP (mg TE/mL)		TPC (mg GAE/mL)	
Run no.	Time (X_1_) (min)	Amplitude (X_2_) (%)	Experimental data	XGBoost predicted	Experimental data	XGBoost predicted	Experimental data	XGBoost predicted
1	14	90	6.90	6.85	60.17	57.92	75.21	73.80
2	12	100	6.48	6.48	54.59	54.60	68.40	68.41
3	12	80	7.02	7.04	62.74	62.16	77.65	77.62
4	12	60	6.57	6.57	58.91	58.94	67.92	67.97
5	8	80	6.57	6.58	52.84	52.86	71.52	71.57
6	12	80	7.05	7.04	62.06	62.16	77.65	77.62
7	10	90	6.70	6.77	54.02	57.27	71.52	73.55
8	16	80	6.76	6.76	52.78	52.74	71.95	71.97
9	12	80	7.05	7.04	61.27	62.16	77.65	77.62
10	10	70	6.87	6.80	61.82	58.84	74.82	72.97
11	14	70	6.81	6.88	56.69	59.49	71.48	73.23
12	12	80	7.02	7.04	62.27	62.16	77.65	77.62
13	12	80	7.05	7.04	62.74	62.16	77.65	77.62
(X optimization parameters)	**12**	**80**	**7.04**		**62.16**		**77.62**	
Experimental values			**7.15 ± 0.03**		**59.80 ± 0.92**		**78.44 ± 1.87**	
% Difference			**0.38**		**1.80**		**0.77**	

a X_1_: time (min); X_2_: amplitude (%); RSM: response surface methodology; TPC: total phenolic content; FRAP: Ferric reducing antioxidant power; mg TE: milligram trolox equivalent; GAE: milligram gallic acid equivalent.

Figure 1 shows the integrated effects of extraction time (8–16 min) and sonication amplitude (60–100%) on total chlorophyll, FRAP, and TPC using reaction surfaces predicted by the XGBoost model. In all panels, a clear ridge line appears to form around 12 min and 80% amplitude. This area marks the optimal treatment window in which all three functional parameters reach their highest values simultaneously. The slope of the duration factor is steeper than that of amplitude, showing a substantial increase in chlorophyll and antioxidant capacity when extending from 8 to 12 min. In contrast, the areas tend to plateau or slightly decrease after 12 min, suggesting that the marginal benefit of prolonged sonication is limited. Similarly, high amplitudes above 90% do not lead to an additional increase in FRAP and TPC, but rather a slight downward trend, likely due to free radical formation from excessive cavitation. The relative flattening of the surface topography in the 12 min/75–85% range indicates that the process has a high operational tolerance in this range and that slight deviations in scale-up do not significantly affect the product quality. Therefore, the combination of a 12 min duration and medium–high (75–85%) amplitude can be recommended as the optimal condition for both chlorophyll stability and overall antioxidant capacity.

**Figure 1:**
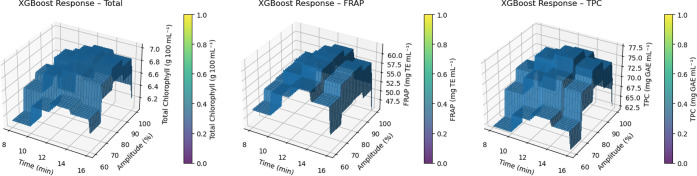
XGBoost predicted response surfaces for ultrasound-assisted extraction of garden cress juice samples. Surfaces show the effect of extraction time (8–16 min) and sonication amplitude (60–100%) on (A) total chlorophyll (g/100 mL), (B) Ferric reducing antioxidant power (FRAP-mg TE/mL), and (C) total phenolic content (TPC-mg GAE/mL). Color bars are min-max normalized (0 = lowest, 1 = highest) for visual comparison between panels. Grid spacing = 0.14 min × 0.68%.

SHAP (Shapley Additive Explanations) value analysis, developed by Lundberg and Lee, is used in XGBoost models to evaluate the contributions of individual variables on the predictions quantitatively and to make the decision mechanism of the model interpretable.^32^ SHAP values measure the contribution of each feature to prediction by evaluating its marginal contribution among all possible feature combinations.^15^ A comparative analysis of the SHAP summary plots for each response variable for the XGBoost model is presented in Figure 2. For total chlorophyll (A), the contributions of both input variables (time and amplitude) to the model are relatively similar in magnitude. Low values of the time variable (blue) have a decreasing effect on the predicted chlorophyll content, while high values of time make a positive contribution. Amplitude, on the other hand, tends to have a positive impact, with amplitude values above 80% increasing the chlorophyll output. Duration is the most influential variable for the FRAP output (B). Low durations led to negative SHAP values, while high durations positively influenced the estimated FRAP value. This confirms the crucial role of extraction duration in the antioxidant capacity. The SHAP plot (C) for TPC shows that both the time and amplitude variables have a broader SHAP distribution. Both variables have both positive and negative effects on performance, so their effects vary depending on the context of the sample. These plots help us to understand the conditions under which the model makes more reliable predictions and aid in optimization decisions.

**Figure 2:**
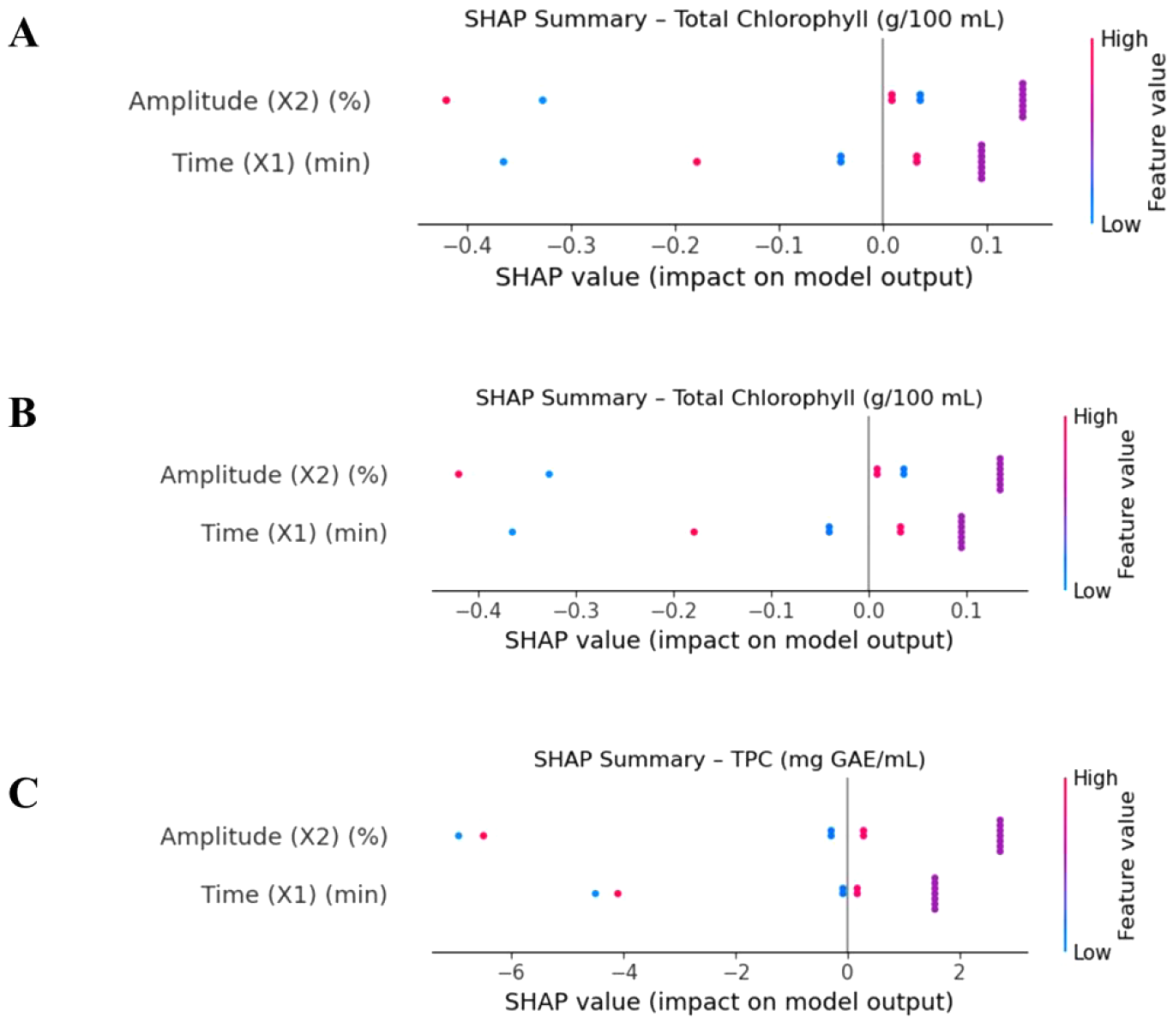
SHAP summary plots for the XGBoost model: (A) total chlorophyll, (B) FRAP, and (C) TPC. The *y*-axis lists the input features—extraction time (min) and sonication amplitude (%)—in descending order of overall importance. Each colored dot represents the SHAP value (impact on model output) of a single observation for the corresponding feature. The horizontal axis shows how strongly that feature pushes the model prediction to higher (right, positive) or lower (left, negative) values. Dot color encodes the raw feature magnitude (low = blue, high = pink/purple), allowing the direction of influence and the underlying feature level to be interpreted simultaneously.

With only 13 test conditions in this study, the statistical significance of the classic performance evaluation with training-test separation is weakened. Therefore, 5-fold cross-validation (CV) was used to investigate the generalization ability of the model. The data are randomly divided into five subsets. Four subsets are used for training in each cycle, and the remaining subset is used as a “hidden” test. Thus, each sample was tested exactly once, and the accuracy measures were calculated as the mean ± standard deviation (SD).

The results presented in Table 2 show that the XGBoost model could not achieve a consistent fitting performance with the limited data set. Although the RMSE value for total chlorophyll (0.214 ± 0.129 g 100 mL^–1^) remained relatively low in absolute terms, the narrow variance range resulted in a strongly negative and unstable *R* ^2^ value (−32.84 ± 59.17). For the FRAP variable, the prediction error (5.93 ± 4.04 mg TE mL^–1^) was practically considerable, and the negative *R* ^2^ value (−13.86 ± 15.15) indicated that the model performed worse than the mean prediction in some areas. The most pronounced inconsistency was observed for the TPC output, where the *R* ^2^ value (−182.84 ± 398.55) and the corresponding RMSE (4.34 ± 2.81 mg GAE mL^–1^) together indicated that the model complexity exceeded the capacity of the available data size and variance structure.

**Table 2:** Cross-Validation Performance Metrics (*R* ^2^ and RMSE) of the XGBoost Model for All Output Variables^a^

Output	*R* ^2^ (5-fold)	RMSE (5-fold)
Total Chlorophyll (g/100 mL)	–32.840 ± 59.165	0.214 ± 0.129
FRAP (mg TE/mL)	–13.857 ± 15.145	5.927 ± 4.044
TPC (mg GAE/mL)	–182.842 ± 398.553	4.342 ± 2.806

a TPC: total phenolic content; FRAP: Ferric reducing antioxidant power; *R* ^2^: coefficient of determination; RMSE: root mean square error; mg TE: milligram trolox equivalent; GAE: milligram gallic acid equivalent. Note: Negative *R* ^2^ values observed during 5-fold cross-validation suggest that the model exhibits poor generalization performance, performing worse than a baseline model predicting the mean response. This outcome likely reflects the limited sample size used for model training.

Taken together, the results show that the XGBoost algorithm can provide very accurate predictions under the conditions of the experimental design (Table 1). For all three results (total chlorophyll, FRAP, and TPC), the relative errors between predicted and experimental values remained below 2%, indicating a reliable predictive capacity within the defined parameter space. The estimated response surfaces (Figure 1) show an optimal range around 12 min and 75–85% amplitude, where the functional results reach their maximum. However, the predictive accuracy of the model decreases beyond this range, indicating a rather context-dependent behavior. This observation is supported by the analysis of the SHAP values (Figure 2), where the influence of amplitude shows a high variability and a nonuniform direction depending on the sample, while time has a more stable and generally positive effect. Despite this promising performance under optimal conditions, the generalizability of the model is restricted by the limited data set, as evidenced by the negative *R* ^2^ values at 5-fold cross-validation (Table 2). For the TPC output, in particular, the high variance of *R* ^2^ indicates instability and potential overfitting. However, when comparing the predictive accuracy of the model with the standard deviations of the experimental measurements (e.g., ±0.92 mg TE/ml for FRAP), it is clear that the variability of the XGBoost output remains within the limits of experimental variation, confirming its practical utility and biological consistency.

### 3.2 Total Bioactive Compounds

The different types of treatments applied had significant effects on the total bioactive compound content of the juice. When the total phenolic content (TPC), total chlorophyll, FRAP (ferric reducing antioxidant power), and β-carotene levels were evaluated, it was observed that the ultrasound-treated samples (UT-GJ) reached the highest values in each parameter (Figure 3). This finding is consistent with the literature reporting that ultrasonic application increases the release of phenolic compounds, thereby enhancing total antioxidant capacity.^33,34^

**Figure 3:**
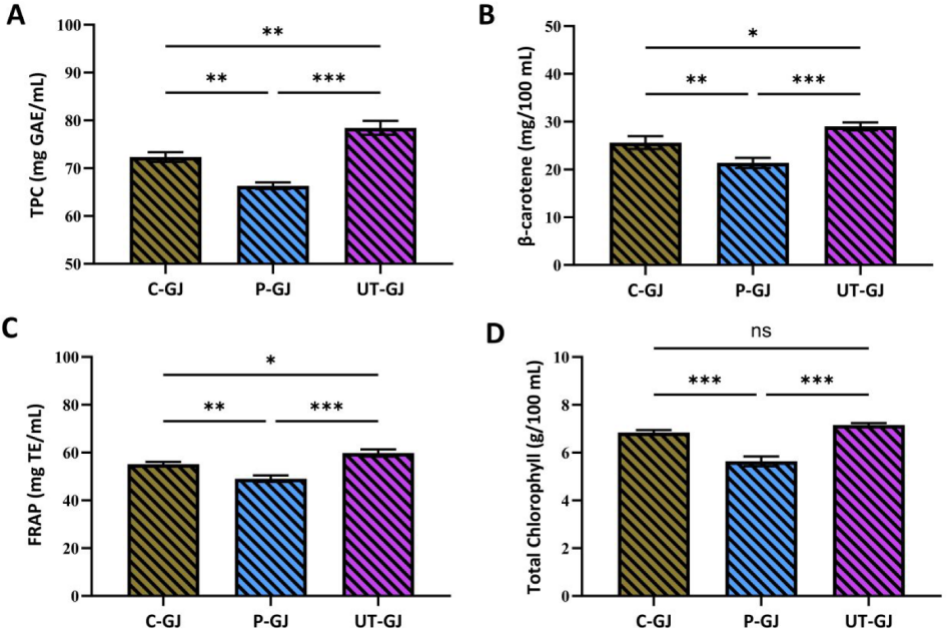
Effects of different treatments (C-GJ: control, P-GJ: pasteurized, UT-GJ: ultrasound-treated) on total phenolic content (TPC-mg GAE/mL) (A), β-carotene (mg/100 mL) (B), FRAP (mg TE/mL) (C), and total chlorophyll (g/100 mL) (D) levels in garden cress juice. Data are presented as mean ± standard deviation (*n* = 3). Different letters indicate statistically significant differences among treatments (*p* < 0.05) according to one-way ANOVA followed by Tukey’s test. Asterisks denote significance levels. ns = not significant (*p* > 0.05); * = *p* < 0.05; ** = *p* < 0.01; *** = *p* < 0.001.

The TPC value was measured at the highest level in the UT-GJ group at 78.44 mg GAE/mL, which was found to be significantly higher than the control group (C-GJ: 72.34 mg GAE/mL) and the pasteurized sample (P-GJ: 66.33 mg GAE/mL). Similarly, in a study conducted on parsley juice, thermosonication also resulted in a significant increase in phenolic compounds such as gallic acid and was found to be more effective than thermal processing.^33^ Similarly, the FRAP value was determined to be 59.80 mg TE/mL in the UT-GJ group, which was significantly different from the control group (55.15 mg TE/mL) and especially from the pasteurized sample (49.07 mg TE/mL). This suggests that the ultrasound treatment led to an increase in the antioxidant capacity. Significant increases in FRAP values were also reported in a recent study on broccoli juice after ultrasound application.^35^

The total chlorophyll content also reached 7.15 g/100 mL in the UT-GJ group, which was significantly higher than the control (6.84 g/100 mL) and pasteurized (5.63 g/100 mL) samples (*p* < 0.01). Finally, the β-carotene level was also measured at the highest value of 29.01 mg/100 mL in the UT-GJ group, and a detrimental effect of pasteurization on this component was observed (P-GJ: 21.39 mg/100 mL; C-GJ: 25.61 mg/100 mL).

Significant correlations were determined among the total bioactive compounds, and these relationships were supported by a Pearson correlation analysis (Figure 4). A robust positive correlation (*r* = 0.98; *p* < 0.01) was found between TPC and FRAP, highlighting the decisive effect of total phenolic compounds on antioxidant capacity. This result was also emphasized in a study on black carrot juice, where it was reported that the increased amount of phenolic compounds after ultrasonic application directly reflected FRAP activity.^34^

**Figure 4:**
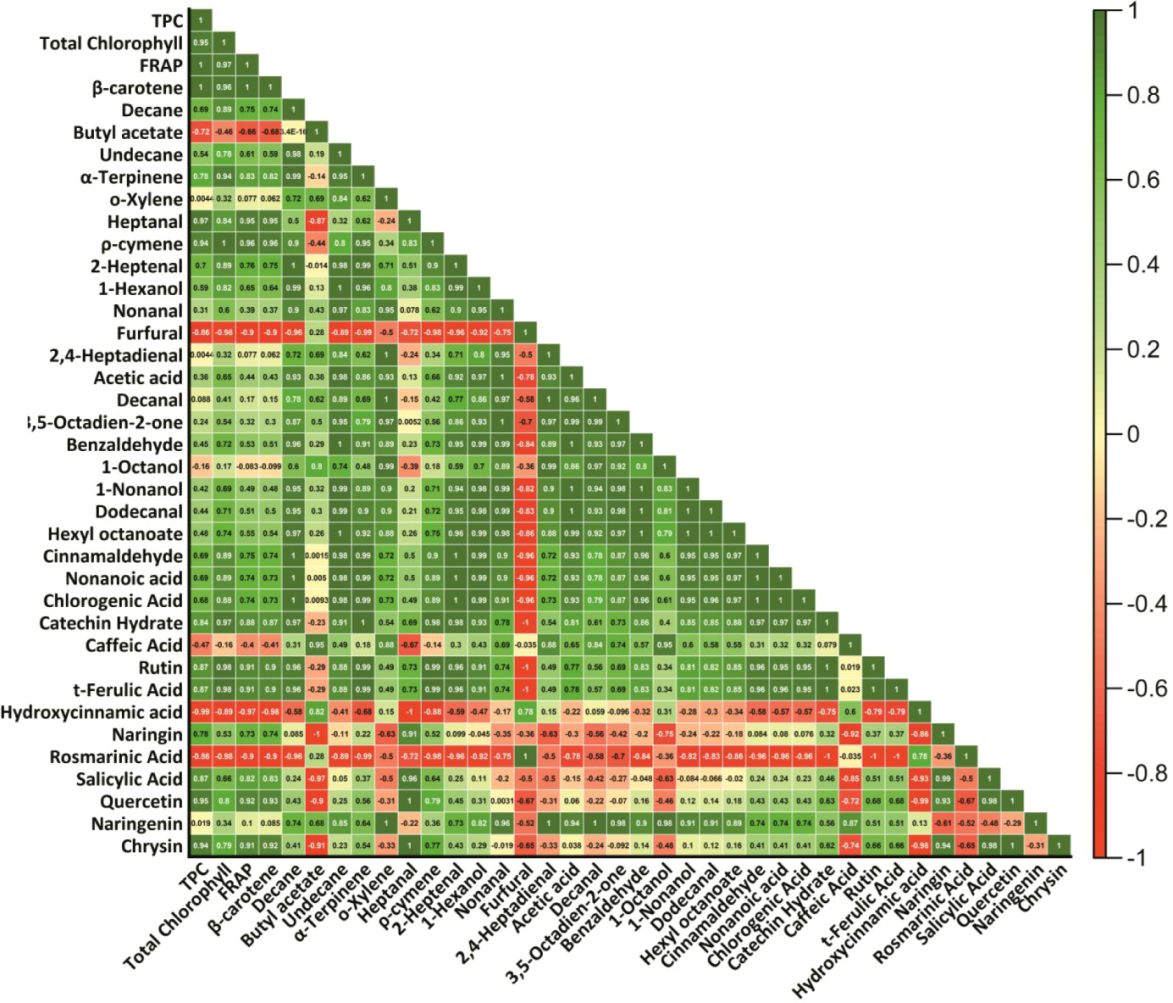
Heatmap showing Pearson correlation coefficients (r) among bioactive compounds in garden cress juices treated with control, pasteurization, and ultrasound methods.

Significant positive correlations were also found between TPC and total chlorophyll (*r* = 0.92) and β-carotene (*r* = 0.95). A high correlation (*r* = 0.93) was found between the FRAP value and β-carotene, indicating that carotenoids also play a role in the antioxidant system. These results demonstrate that the ultrasound process not only preserves phenolic compounds but also releases other bioactive compounds at a high rate. In contrast, the pasteurization process caused a decrease in heat-sensitive compounds such as chlorophyll and carotenoids, leading to a reduction in total antioxidant capacity. In another study conducted by Marinaccio et al. (2025), it was observed that ultrasound-assisted extraction application provided the highest levels of total phenolic content (TPC), total flavonoid content (TFC), and antioxidant capacity in grape seed extracts.^36^ The findings clearly demonstrate that nonthermal technologies (particularly ultrasound and thermosonication) are more advantageous than traditional thermal processes in preserving bioactive components.^33,35^

### 3.3 Phenolic Profile

The phenolic compound profile of cress juice varied significantly, depending on the types of treatments applied (Table 3). A total of 12 different phenolic compounds were determined quantitatively; among these compounds, chrysin, chlorogenic acid, quercetin, catechin hydrate, t-ferulic acid, and naringin were particularly prominent. Chlorogenic acid was found at high levels in both the control (29.82 ± 2.22 mg/L) and ultrasound groups (28.69 ± 2.65 mg/L), and at lower levels in the pasteurized sample (25.98 ± 1.58 mg/L). However, the difference between the groups was not found to be statistically significant (*p* > 0.05). In contrast, pasteurization caused substantial losses in heat-labile compounds such as catechin hydrate (P-GJ: 0.11 ± 0.01a mg/L; C-GJ: 0.71 ± 0.03b; UT-GJ: 0.68 ± 0.12b; *p* < 0.05). Ferulic acid also decreased with pasteurization but was maintained at levels similar to the control group under ultrasound application. Naringin and salicylic acid were detected only in the ultrasound group, indicating that ultrasound application is practical in converting these compounds from bound forms to free forms. Pasteurization-induced increases were observed in compounds such as rosmarinic acid, suggesting this may result from the transformation of some compounds by heat treatment. Similarly, in another study conducted on Jabuticaba juice, low-, medium-, and high-intensity ultrasound applications were compared with thermal pasteurization; it was observed that high-intensity ultrasound, in particular, increased the amounts of TPC and anthocyanins, as well as increased the levels of ellagic acid, gallic acid, and flavonoids. This increase is associated with the enhanced extraction of phenolic compounds due to mechanical and thermal effects on the cellular matrix. However, thermal pasteurization has been reported to cause losses in these heat-sensitive compounds by causing their degradation through thermal hydrolysis and oxidation.^37^

**Table 3:** Changes in Total Phenolic Compounds of Garden Cress Samples Subjected to Different Treatments (C-GJ, P-GJ, and UT-GJ)[Table-fn tbl3fn1]

	Samples		
Phenolic Compounds (μg/mL)	C-GJ	P-GJ	UT-GJ
Chlorogenic Acid	29.82 ± 2.22^a^	25.98 ± 1.58^a^	28.69 ± 2.65^a^
Catechin Hydrate	0.71 ± 0.03^b^	0.11 ± 0.01^a^	0.68 ± 0.12^b^
Caffeic Acid	19.08 ± 0.79^a^	17.16 ± 1.04^a^	15.46 ± 1.03^a^
Rutin	7.54 ± 0.22^a^	7.02 ± 0.43^a^	7.55 ± 1.10^a^
t-Ferulic Acid	6.11 ± 0.22^b^	4.01 ± 0.24^a^	6.14 ± 0.64^b^
Hydroxycinnamic acid	5.23 ± 0.21^ab^	5.84 ± 0.43^b^	4.18 ± 0.38^a^
Naringin	0.00 ± 0.00^a^	0.22 ± 0.02^a^	1.31 ± 0.12^b^
Rosmarinic Acid	0.22 ± 0.01^a^	0.44 ± 0.03^b^	0.22 ± 0.02^a^
Salicylic Acid	0.00 ± 0.00^a^	0.00 ± 0.00^a^	0.40 ± 0.03^b^
Quercetin	1.77 ± 0.10^a^	1.60 ± 0.10^a^	2.39 ± 0.16^b^
Naringenin	2.32 ± 0.10^b^	1.21 ± 0.07^a^	1.24 ± 0.08^a^
Chrysin	34.25 ± 3.17^b^	0.00 ± 0.00^a^	176.51 ± 7.72^c^

i C-GJ: control garden cress juice; P-GJ: thermal pasteurized garden cress juice; UT-GJ: ultrasound-treated garden cress juice. Data are presented as mean ± standard deviation (*n* = 3). Different superscript letters within the same row indicate statistically significant differences among treatments (*p* < 0.05; one-way ANOVA followed by Tukey’s test).

Relationships between phenolic compounds and bioactive parameters were detailed by a Pearson correlation analysis (Figure 4). Chrysin showed a remarkable increase in ultrasound (UT-GJ: 176.51 ± 7.72c mg/L; C-GJ: 34.25 ± 3.17b; P-GJ: 0.00 ± 0.00a; *p* < 0.001), and this compound was found to have a robust positive correlation with TPC (*r* = 0.94) and FRAP (*r* = 0.97) (*p* < 0.01). These results demonstrate that chrysin contributes significantly to antioxidant capacity. Similarly, quercetin was increased dramatically by ultrasound application (*p* < 0.05) and positively correlated with FRAP (*r* = 0.89). Positive correlations were observed between catechin hydrate and both FRAP (*r* = 0.86) and TPC (*r* = 0.81). These correlations support the decisive role of phenolic compounds in antioxidant activity. In addition, the high levels of compounds such as naringin and cinnamaldehyde detected only in the UT-GJ group indicate that ultrasound technology facilitates structural dissolution, allowing bound phenolic compounds to become free. In a study conducted on pineapple juice, it was reported that ultrasound application facilitated the release of intracellular phenolic compounds by breaking down cell walls, thus providing a significant increase in TPC content and antioxidant capacity. On the other hand, it has been stated that thermal pasteurization negatively affects various quality parameters, especially TPC, and causes deterioration.^38^ Similarly, in another study conducted on a mixture of carrot and orange juice, ultrasound treatment creates microbubbles in the liquid medium through acoustic cavitation, causing them to collapse. This mechanism has the potential to increase TPC, aroma compounds, and antioxidant capacity by breaking down fruit tissues and cell walls. In contrast, pasteurization may cause losses in these properties due to high temperatures.^39^

Various studies have shown that ultrasound technology is considered a “green” technology because it is an environmentally friendly processing method due to its advantages, such as higher product quality, energy savings, and minimum chemical use, while it also draws attention as a practical approach in preserving nutrients and increasing bioaccessibility.^40,41^ In general, ultrasound application stands out as an effective nonthermal method that improves both nutritional and functional quality by maintaining and/or enhancing the release of phenolic compounds. Pasteurization caused losses in some important phenolic compounds and reduced the antioxidant capacity of the product.

### 3.4 Volatile Aroma Compounds

The volatile compound profile determined in garden cress juice showed significant changes depending on the type of treatment applied (Table 4). A total of 22 aroma compounds, including 8 aldehydes, one ketone, two acids, three terpene compounds, two esters, 3 alcohols, and three other compounds, were detected in garden cress water samples. At the same time, the highest amount of aroma compounds was 72.71 μg/kg in the C-GJ sample and 38.63 and 54.42 μg/kg in the P-GJ and UT-BGJ samples, respectively. The most abundant aroma compounds in garden cress water samples were 1-hexanol, benzaldehyde, cinnamaldehyde, nonanal, and undecane. Similar to our results, Abdallah et al. (2020) reported that predominant aroma compounds in garden cress samples were 1-hexanol, nonanal, and cinnamaldehyde.^42^ Particularly, 1-hexanol was found at high levels in the control (23.8 μg/kg) and ultrasound-treated (20.02 μg/kg) groups. In comparison, it showed a statistically significant decrease in the pasteurized sample (14.4 μg/kg) (*p* < 0.05). Similarly, the benzaldehyde amount, while highest in the control sample (15.74 μg/kg), was partially preserved with ultrasound treatment (11.22 μg/kg) and significantly decreased with pasteurization (7.39 μg/kg) (*p* < 0.05). These decreases could be explained by the decomposition or evaporation of heat-sensitive volatile compounds under high temperatures. Furthermore, some compounds, such as furfural, were found only in the pasteurized sample, suggesting that this treatment produces new aroma compounds specific to heat treatments such as the Maillard reaction. The same results for mandarin (Citrus unshiu) juice were reported by Cheng et al. (2020), while similar results for pomegranate juice were found by Yıkmış et al. (2022).^43,44^ The pasteurization and thermosonication treatments led to the disappearance of some volatile compounds. Previous works reported identical results.^29,45^ The only ketone compound that was detected in garden cress juice was 3,5-Octadien-2-one. The C-GJ sample had the highest amount of 3,5-Octadien-2-one, with a detection of 1.43 μg/kg. The UT-GJ and P-GJ samples had the following highest amounts, with detections of 0.67 and 0.41 μg/kg, respectively. Two ester compounds, Butyl acetate and Hexyl octanoate, were detected in all garden cress juice samples. α-terpinene, ρ-cymene, and *ο*-xylene were recovered from samples. Bahrami et al. (2016) reported that the major constituents of the oil of *L. sativum* of Iran, isolated by hydrodistillation, were monoterpenes such as *p*-cymene (1.67%), myrcene (2.90%), and α-thujene (88.86%).^46^ Strong relationships were identified between the aroma profile and bioactive compounds (Figure 4). In particular, a significant positive correlation was found between 1-hexanol and TPC (*r* = 0.87; *p* < 0.05), indicating that phenolic compounds may help preserve aroma compounds by contributing to oxidative stability. Similarly, benzaldehyde showed a strong positive correlation with both FRAP values (*r* = 0.91; *p* < 0.05) and chlorophyll content (*r* = 0.89; *p* < 0.05). This indicates that the antioxidant capacity associated with phenolic structures may play a role in protecting aroma compounds against oxidation. Additionally, positive correlations were observed between cinnamaldehyde and TPC (*r* = 0.83) and β-carotene (*r* = 0.88), supporting the preservation of both aroma and functional qualities of this compound after ultrasound treatment.

**Table 4:** Determination of Volatile Profiles of C-GJ, P-GJ, and UT-GJ[Table-fn tbl4fn1]

Compound	RI	C-GJ (μg/kg)	P-GJ (μg/kg)	UT-GJ (μg/kg)
Decane	1010	0.21 ± 0.06^b^	0.00 ± 0.00^a^	0.15 ± 0.05^ab^
Butyl acetate	1072	0.31 ± 0.07^a^	0.30 ± 0.05^a^	0.27 ± 0.02^a^
Undecane	1098	1.36 ± 0.11^c^	0.55 ± 0.10^a^	0.99 ± 0.04^b^
α-Terpinene	1170	0.64 ± 0.08^a^	0.31 ± 0.08^a^	0.59 ± 0.23^a^
*o*-Xylene	1190	0.18 ± 0.01^b^	0.00 ± 0.00^a^	0.00 ± 0.00^a^
Heptanal	1192	0.31 ± 0.06^a^	0.29 ± 0.11^a^	0.36 ± 0.03^a^
ρ-cymene	1276	0.13 ± 0.03^b^	0.00 ± 0.00^a^	0.16 ± 0.01^b^
2-Heptenal	1344	0.22 ± 0.06^b^	0.00 ± 0.00^a^	0.16 ± 0.06^ab^
1-Hexanol	1355	23.8 ± 0.87^b^	14.4 ± 1.33^a^	20.02 ± 0.70^b^
Nonanal	1397	3.58 ± 0.37^b^	0.94 ± 0.12^a^	1.79 ± 0.18^a^
Furfural	1464	0.00 ± 0.00^a^	0.22 ± 0.04^b^	0.00 ± 0.00^a^
2,4-Heptadienal	1470	0.24 ± 0.04^b^	0.00 ± 0.00^a^	0.00 ± 0.00^a^
Acetic acid	1472	5.12 ± 0.71^a^	3.51 ± 0.08^a^	4.11 ± 0.49^a^
Decanal	1496	2.42 ± 0.62^a^	1.24 ± 0.36^a^	1.36 ± 0.19^a^
3,5-Octadien-2-one	1525	1.43 ± 0.22^b^	0.41 ± 0.10^a^	0.67 ± 0.17^a^
Benzaldehyde	1540	15.74 ± 1.15^b^	7.39 ± 0.84^a^	11.22 ± 1.03^a^
1-Octanol	1562	3.81 ± 0.61^b^	2.26 ± 0.33^ab^	1.94 ± 0.12^a^
1-Nonanol	1658	6.37 ± 0.54^b^	4.59 ± 0.33^a^	5.35 ± 0.23^ab^
Dodecanal	1716	0.61 ± 0.17^b^	0.00 ± 0.00^a^	0.27 ± 0.05^ab^
Hexyl octanoate	1794	0.47 ± 0.13^a^	0.18 ± 0.03^a^	0.32 ± 0.09^a^
Cinnamaldehyde	2024	4.74 ± 0.54^b^	1.64 ± 0.16^a^	3.85 ± 0.24^b^
Nonanoic acid	2162	1.02 ± 0.09^b^	0.40 ± 0.09^a^	0.84 ± 0.18^ab^

i RI: retention index; n.d.: not determined; C-GJ: Control garden cress juice; P-GJ: thermal pasteurized garden cress juice; UT-GJ: ultrasound-treated garden cress juice. Results are presented as mean ± standard deviation (*n* = 3). Values with the different letters on the same line are significantly different (*p* < 0.05).

### 3.5 Bioaccessibility and Digestive Stability

The effects of different treatments applied to cress juice on the durability and bioaccessibility of bioactive components during the digestive process have yielded remarkable results (Table 5). In the undigested phase, the highest values for all bioactive components were observed in the ultrasound-treated sample (UT-GJ); this was significant (*p* < 0.05) for total phenolic matter (78.44 ± 1.47 mg GAE/mL), antioxidant capacity (FRAP: 59.80 ± 1.52 mg TE/mL), and total chlorophyll (7.15 ± 0.08 g/100 mL). These values are significantly lower in pasteurized samples (P-GJ), indicating that heat treatments negatively affect the stability of phenolic structures and chlorophylls. At the beginning of the oral digestion phase, a significant decrease in the levels of bioactive compounds was observed in all groups, indicating that these compounds were not stable at this stage. However, UT-GJ samples stood out with significantly higher TPC (54.93 ± 2.11 mg GAE/mL), FRAP (41.54 ± 1.50 mg TE/mL), and chlorophyll (5.05 ± 0.06 g/100 mL) values compared to the other groups (*p* < 0.05). A similar trend continued in the gastric phase, with the UT-GJ group showing higher phenolic (40.35 ± 1.84 mg GAE/mL) and antioxidant capacity (30.29 ± 1.11 mg TE/mL) compared to C-GJ and P-GJ. A study comparing high-intensity ultrasound, freeze-drying, and pasteurization processing methods on blueberries showed that pasteurization maintained high stability of TPC content, phenolic profile, and antioxidant capacity in the gastric phase. At the same time, these values decreased in the intestinal phase. High-intensity ultrasound increased these components after the gastric phase and positively changed the structure of the food matrix through mechanisms such as cavitation. This study also reported that the positive effect was positively correlated with the increase in power density of ultrasound administration.^47^ In a similar study, it was observed that thermal pasteurization applied to mixed vegetable juice caused a significant decrease in total phenolic compound (TPC) content and antioxidant capacity during the in vitro gastric digestion phase; whereas, ultrasonic treatment maintained the stability of phenolic compounds and improved antioxidant capacity by increasing bioavailability. Additionally, this study found an increase in TPC levels in the intestinal phase. These findings support the idea that heat treatments can negatively impact the physical and chemical properties of food, as well as its heat-sensitive bioactive compounds.^48^ These results show that cell walls are broken down by the ultrasound process, resulting in the creation of phenolic structures that are more resistant to the digestive process, thus increasing bioaccessibility. In the small intestine (intestinal) phase, bioactive compound levels were lowest in all groups due to the natural effects of digestion. However, the UT-GJ sample managed to maintain the highest values even in this phase: TPC 27.43 ± 1.25 mg GAE/mL, FRAP 17.20 ± 1.00 mg TE/mL, and total chlorophyll 2.32 ± 0.18 g/100 mL were found to be significantly different from the other groups (*p* < 0.05). When evaluated in terms of total recovery rates (%), the results were 34.96% for TPC, 32.50% for chlorophyll, and 28.81% for FRAP in the UT-GJ sample. This high recovery rate obtained for TPC indicates that ultrasound treatment provides greater preservation of phenolic compounds in the digestive tract and increases bioaccessibility. According to Pearson correlation analysis, the positive and significant correlation between TPC and FRAP was preserved in the intestinal phase (*r* = 0.93; *p* < 0.01), indicating that phenolic compounds continue to contribute to antioxidant activity after digestion. Some studies that examined ultrasound and pasteurization processes separately have yielded remarkable findings. Ozcan and Damar (2023) reported that ultrasound significantly improved the bioavailability of phenolic compounds, particularly in the stomach phase, in their study evaluating spinach roots. Due to ultrasound’s ability to preserve sensitive compounds, it is considered a promising extraction technique, providing higher yields for heat-sensitive phytochemicals such as chlorophyll.^49^ On the other hand, it was stated that pasteurization applied to a smoothie containing strawberries, oranges, apples, and bananas had no significant effect on the phenolic compound profile, antioxidant capacity, bioaccessibility, and digestion parameters. It was emphasized that these results are related to the structural properties of the food matrix used and the application of pasteurization at low temperatures; also, attention was drawn to the interest in new-generation technologies, such as ultrasound.^50^ Additionally, another study evaluating both conventional thermal pasteurization (high temperature, short time) and nonthermal high-pressure pasteurization reported that both methods were able to preserve TPC levels. In terms of the digestive process, thermal pasteurization significantly increased the bioaccessibility of the polyphenols. This effect could be explained by the softening and breakdown of cell walls, which allows these compounds to be more easily released from the matrix.^51^ However, solely on the basis of the results of this study, it would not be scientifically generalizable to conclude that different classes of pasteurization techniques are more effective than ultrasound application. In addition, the study conducted by Roobab et al. (2023) stated that ultrasound application improves the fruit juice quality. Unlike thermal processes, it has the potential to preserve and increase bioactive components and can also increase bioaccessibility by providing easier digestion and absorption at the cellular level.^52^ Ultrasound treatment was found to increase the amount of bioactive components and significantly improve their stability and bioaccessibility during the digestive process. In contrast, pasteurization decreased the total recovery rate by increasing the loss of bioactive compounds in the digestion phases.

**Table 5:** Changes in Total Chlorophyll, Ferric Reducing Antioxidant Power (FRAP), and Total Phenolic Content (TPC) of Garden Cress Juice Samples Subjected to Different Treatments (C-GJ, P-GJ, and UT-GJ)[Table-fn tbl5fn1]

Phases	Samples	Total Chlorophyll (g/100 mL)	FRAP (mg TE/mL)	TPC (mg GAE/mL)
Undigested	C-GJ	6.84 ± 0.11^b^	55.15 ± 0.99^b^	72.34 ± 1.02^b^
	P-GJ	5.63 ± 0.20^a^	49.07 ± 1.36^a^	66.33 ± 0.70^a^
	UT-GJ	7.15 ± 0.08^b^	59.80 ± 1.52^c^	78.44 ± 1.47^c^
Oral Digestion	C-GJ	4.60 ± 0.07^b^	37.08 ± 0.67^b^	48.64 ± 0.68^b^
	P-GJ	3.74 ± 0.13^a^	32.58 ± 0.90^a^	44.04 ± 0.46^a^
	UT-GJ	5.05 ± 0.06^c^	41.54 ± 1.50^c^	54.93 ± 2.11^c^
Gastric Digestion	C-GJ	3.22 ± 0.05^b^	25.95 ± 0.47^b^	33.38 ± 1.33^b^
	P-GJ	2.49 ± 0.08^a^	21.19 ± 1.69^a^	28.51 ± 1.43^a^
	UT-GJ	3.68 ± 0.04^c^	30.29 ± 1.11^c^	40.35 ± 1.84^c^
Intestinal Digestion	C-GJ	2.02 ± 0.04^b^	15.81 ± 0.94^b^	23.15 ± 0.33^b^
	P-GJ	1.42 ± 0.24^a^	12.63 ± 1.18^a^	17.47 ± 0.84^a^
	UT-GJ	2.32 ± 0.18^b^	17.20 ± 1.00^b^	27.43 ± 1.25^c^
Recovery %	C-GJ	29.59 ± 0.11^ab^	28.67 ± 1.63^a^	32.00 ± 0.00^b^
	P-GJ	25.21 ± 3.45^a^	25.79 ± 3.15^a^	26.35 ± 1.48^a^
	UT-GJ	32.50 ± 2.78^b^	28.81 ± 2.34^a^	34.96 ± 1.02^c^

i C-GJ: Control garden cress juice; P-GJ: thermal pasteurized garden cress juice; UT-GJ: ultrasound-treated garden cress juice; FRAP: Ferric reducing antioxidant power; mg TE: milligram trolox equivalent; TPC: total phenolic content; mg GAE: milligram gallic acid equivalent.

### 3.6 Discrimination of Samples via Multivariate Clustering

Multivariate statistical analyses are crucial tools for the detailed classification and interpretation of variations in the chemical and functional properties of processed food products. The PCA score plot (Figure 5) obtained in this study separated control (C-GJ), pasteurized (P-GJ), and ultrasound-treated (UT-GJ) cress juice samples based on their bioactive compound profiles. PC1 and PC2, in particular, account for a significant portion of the total variance, indicating that the treatments substantially affect the distribution of sample components. The clustering of UT-GJ samples along the positive PC1 axis supports the effectiveness of ultrasound treatment in maintaining or enhancing functional elements, such as phenolic compounds, chlorophyll, and β-carotene. Conversely, the more compact clustering of pasteurized samples along the negative PC1 axis suggests that heat processing leads to a reduction in some bioactive compounds. Overall, the PCA analysis not only visualizes the separation but also reveals that the relationships between the samples are statistically significant at the level of the principal components.

**Figure 5:**
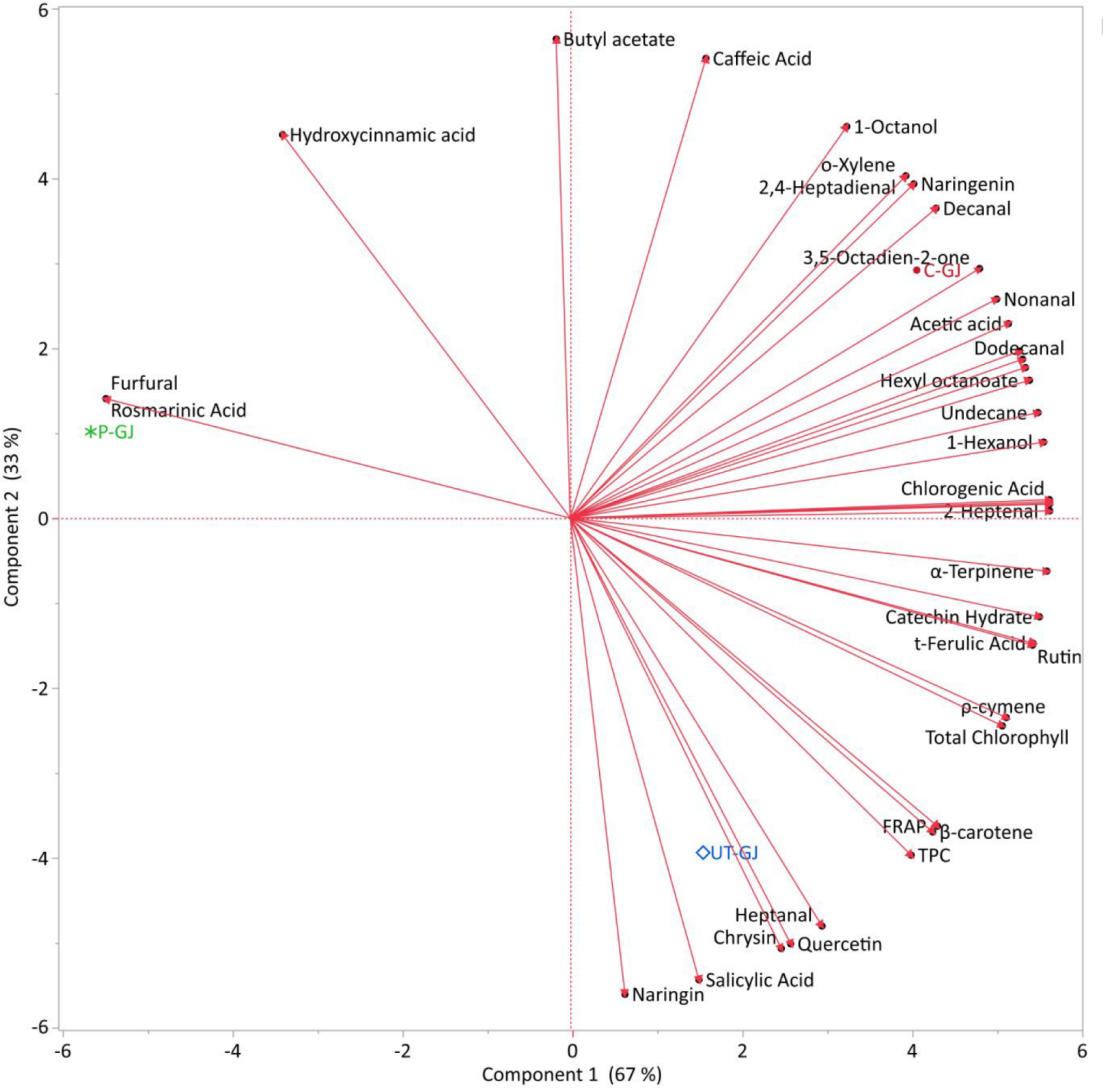
Principal Component Analysis (PCA1 and PCA2) score plot showing the distribution of garden cress juice samples (C-GJ: control, P-GJ: pasteurized, UT-GJ: ultrasound-treated) based on their bioactive compound profiles.

This distinction is supported by the hierarchical clustering dendrogram shown in Figure 5A. Specifically, in the dendrogram generated using the Ward method and Euclidean distance metric, UT-GJ samples clustered separately, while P-GJ and C-GJ samples grouped more closely. This structure highlights the extent of chemical profile differences caused by the ultrasound treatment of the samples. The constellation plot in Figure 5-B visually reinforces the relationships among the samples; the unique spatial position of the UT-GJ samples indicates that they gained a biochemical identity distinct from the other treatments. This clearly underscores the importance of analyzing multivariate data and shows that the effects of food processing techniques on product quality should be assessed by using comprehensive data sets rather than just individual measurements. In summary, these multivariate visualization and classification results demonstrate that nonthermal technologies, especially ultrasound, are effective and reliable tools for advanced analysis of how the structures of functional food ingredients are affected. Multivariate cluster analysis based on bioactive compound profiles of garden cress juice samples is shown in Figure 6.

**Figure 6:**
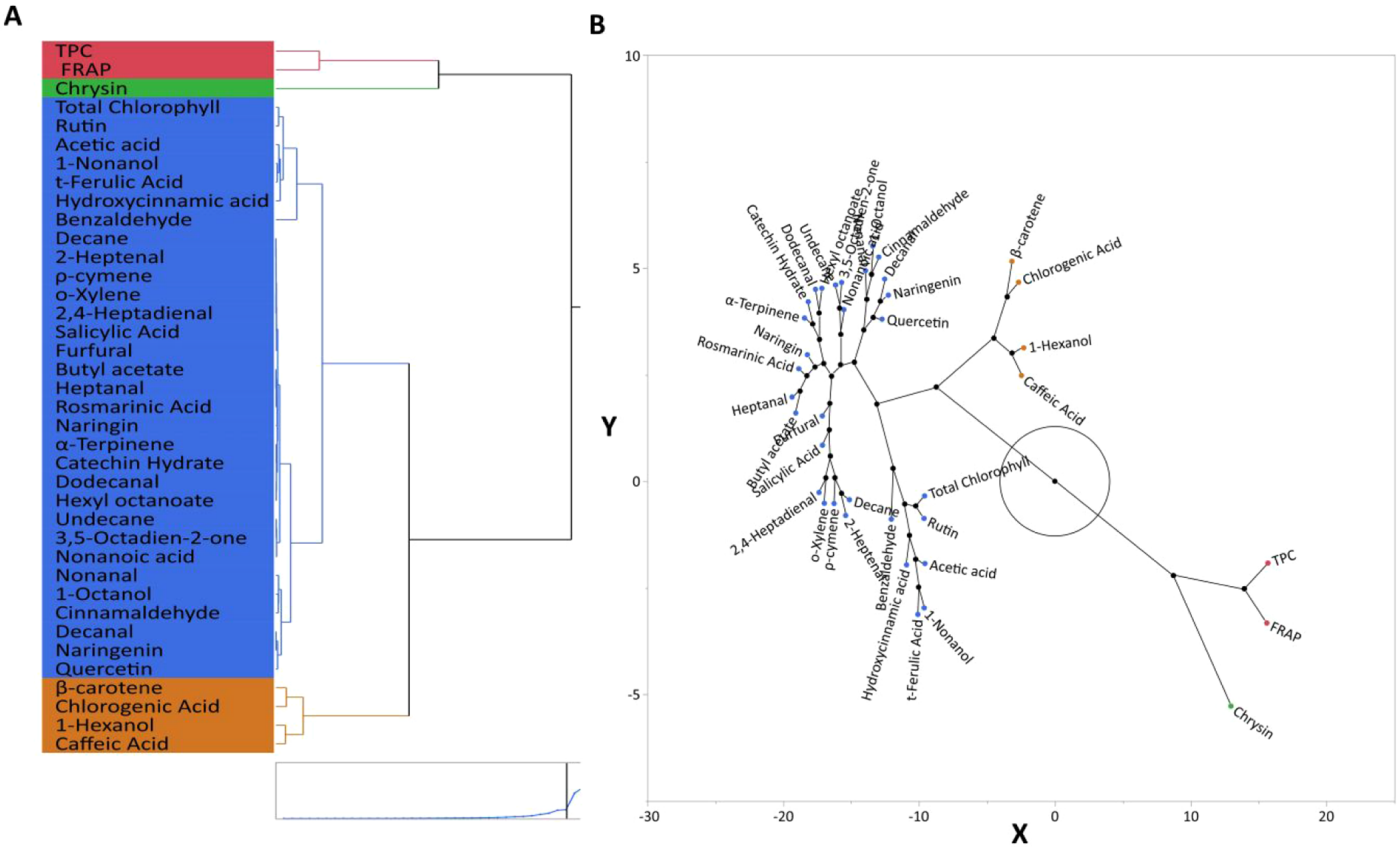
Multivariate clustering analysis of garden cress juice samples (C-GJ: control; P-GJ: pasteurized; UT-GJ: ultrasound-treated) based on their bioactive compound profiles. Panel A shows the hierarchical clustering dendrogram constructed using Ward’s method and Euclidean distance, revealing treatment-based grouping patterns. Panel B shows the constellation plot, illustrating the similarity relationships among samples (arbitrary units) and visually confirming the separation trends observed in the clustering analysis.

## 4 Conclusions

This study showed that ultrasound technology effectively enhances the functional and nutritional qualities of garden cress (*Lepidium sativum*) juice. The process, optimized with the XGBoost algorithm, achieved high accuracy. Under optimal conditions (12 min, 80% amplitude) identified by the Box–Behnken experimental design, levels of total phenolics (78.44 mg GAE/mL), total chlorophyll (7.15 g/100 mL), and antioxidant capacity (FRAP: 59.80 mg TE/mL) increased significantly compared to control and pasteurized samples (*p* < 0.05). HPLC-DAD analysis revealed that ultrasound enriched the phenolic profile by releasing chrysin, quercetin, and other phenolic compounds. GC-MS results showed that aroma compounds, especially 1-hexanol, benzaldehyde, and cinnamaldehyde, were largely preserved. An in vitro digestion simulation indicated a significant rise in bioaccessibility in ultrasound-treated samples, with total postdigestion recovery rates of 34.96% for TPC, 32.50% for chlorophyll, and 28.81% for FRAP. PCA and clustering analyses confirmed that ultrasound treatment distinguished the samples biochemically. These findings demonstrate that combining ultrasound with AI-based optimization provides an effective strategy for preserving bioactive compounds, maintaining flavor integrity, and supporting the sustainable industrial production of functional foods.
